# Deciphering the Evolution of Cephalosporin Resistance to Ceftolozane-Tazobactam in Pseudomonas aeruginosa

**DOI:** 10.1128/mBio.02085-18

**Published:** 2018-12-11

**Authors:** Melissa D. Barnes, Magdalena A. Taracila, Joseph D. Rutter, Christopher R. Bethel, Ioannis Galdadas, Andrea M. Hujer, Emilia Caselli, Fabio Prati, John P. Dekker, Krisztina M. Papp-Wallace, Shozeb Haider, Robert A. Bonomo

**Affiliations:** a Research Service, Louis Stokes Cleveland Department of Veterans Affairs, Cleveland, Ohio, USA; b Department of Medicine, Case Western Reserve University, Cleveland, Ohio, USA; c Department of Molecular Biology and Microbiology, Case Western Reserve University, Cleveland, Ohio, USA; d Department of Pharmacology, Case Western Reserve University, Cleveland, Ohio, USA; e Department of Biochemistry, Case Western Reserve University, Cleveland, Ohio, USA; f Department of Proteomics and Bioinformatics, Case Western Reserve University, Cleveland, Ohio, USA; g Department of Life Science, University of Modena and Reggio Emilia, Modena, Italy; h Department of Laboratory Medicine, Clinical Center, Microbiology Service, National Institutes of Health, Bethesda, Maryland, USA; i UCL School of Pharmacy, University College London, London, United Kingdom; j CWRU-Cleveland VAMC Center for Antimicrobial Resistance and Epidemiology (Case VA CARES), Cleveland, Ohio, USA; k Geriatric Research Education and Clinical Centers (GRECC), Louis Stokes Cleveland Department of Veterans Affairs, Cleveland, Ohio, USA; Lahey Hospital and Medical Center

**Keywords:** AmpC, PDC-3, antibiotic resistance, beta-lactam, beta-lactamase, ceftolozane, omega loop

## Abstract

Pseudomonas aeruginosa produces a class C β-lactamase (e.g., PDC-3) that robustly hydrolyzes early generation cephalosporins often at the diffusion limit; therefore, bacteria possessing these β-lactamases are resistant to many β-lactam antibiotics. In response to this significant clinical threat, ceftolozane, a 3′ aminopyrazolium cephalosporin, was developed. Combined with tazobactam, ceftolozane promised to be effective against multidrug-resistant P. aeruginosa. Alarmingly, Ω-loop variants of the PDC β-lactamase (V213A, G216R, E221K, E221G, and Y223H) were identified in ceftolozane/tazobactam-resistant P. aeruginosa clinical isolates. Herein, we demonstrate that the Escherichia coli strain expressing the E221K variant of PDC-3 had the highest minimum inhibitory concentrations (MICs) against a panel of β-lactam antibiotics, including ceftolozane and ceftazidime, a cephalosporin that differs in structure largely in the R2 side chain. The *k*_cat_ values of the E221K variant for both substrates were equivalent, whereas the *K_m_* for ceftolozane (341 ± 64 µM) was higher than that for ceftazidime (174 ± 20 µM). Timed mass spectrometry, thermal stability, and equilibrium unfolding studies revealed key mechanistic insights. Enhanced sampling molecular dynamics simulations identified conformational changes in the E221K variant Ω-loop, where a hidden pocket adjacent to the catalytic site opens and stabilizes ceftolozane for efficient hydrolysis. Encouragingly, the diazabicyclooctane β-lactamase inhibitor avibactam restored susceptibility to ceftolozane and ceftazidime in cells producing the E221K variant. In addition, a boronic acid transition state inhibitor, LP-06, lowered the ceftolozane and ceftazidime MICs by 8-fold for the E221K-expressing strain. Understanding these structural changes in evolutionarily selected variants is critical toward designing effective β-lactam/β-lactamase inhibitor therapies for P. aeruginosa infections.

## Abstract (precis)

The presence of β-lactamases (e.g., PDC-3) that have naturally evolved and acquired the ability to break down β-lactam antibiotics (e.g., ceftazidime and ceftolozane) leads to highly resistant and potentially lethal Pseudomonas aeruginosa infections. We show that wild-type PDC-3 β-lactamase forms an acyl enzyme complex with ceftazidime, but it cannot accommodate the structurally similar ceftolozane that has a longer R2 side chain with increased basicity. A single amino acid substitution from a glutamate to a lysine at position 221 in PDC-3 (E221K) causes the tyrosine residue at 223 to adopt a new position poised for efficient hydrolysis of both cephalosporins. The importance of the mechanism of action of the E221K variant, in particular, is underscored by its evolutionary recurrences in multiple bacterial species. Understanding the biochemical and molecular basis for resistance is key to designing effective therapies and developing new β-lactam/β-lactamase inhibitor combinations.

## INTRODUCTION

Pseudomonas aeruginosa infections are among the most serious health threats of this century, mostly hospital acquired by immunocompromised patients. P. aeruginosa is responsible for approximately 51,000 nosocomial infections every year in the United States (1), and it is one of the top three pathogens of global concern specifically addressed by the World Health Organization in their guidelines for prevention and control released late in 2017 (2). In a survey that identified more than 400,000 drug-resistant pathogens collected from 4,515 U.S. hospitals between 2011 and 2014, P. aeruginosa was the 6th most common nosocomial pathogen, ranking 2nd in ventilator-associated pneumonia and 3rd in catheter-associated urinary tract infections (3). Particularly notorious in cystic fibrosis, P. aeruginosa infects 80% of patients by 18 years of age and results in a 2.6-fold increase in mortality. Alarming levels of resistance are emerging without compensatory therapies to overcome these infections. P. aeruginosa produces a chromosomally encoded class C cephalosporinase (*Pseudomonas*-derived cephalosporinase, PDC β-lactamase) that is often responsible for high-level resistance to β-lactam antibiotics.

**FIG 1:**
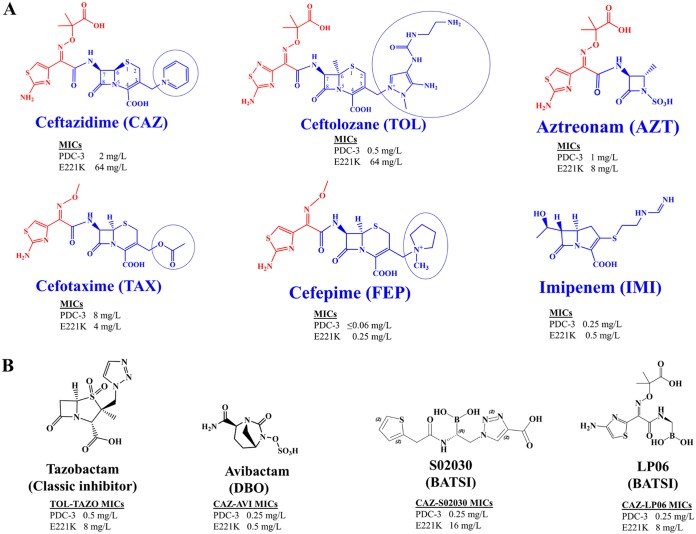
Structures of β-lactam antibiotics (A) and inhibitors (B) tested in the MIC assays. The R1 side chains of the β-lactam antibiotics are shown in red, and the R2 side chains of the cephalosporins are circled. MIC values from Table 1 are listed for PDC-3 versus E221K.

Ceftolozane, a “fifth-generation” cephalosporin, is characterized by a 3′ aminopyrazolium substitution in the R2 side chain (Fig. 1) and is commercially partnered with tazobactam, a penicillin sulfone and irreversible β-lactamase inhibitor. The ceftolozane-tazobactam combination is active against P. aeruginosa isolates resistant to ceftazidime, carbapenems, and piperacillin-tazobactam. The advantage of the ceftolozane-tazobactam combination is that ceftolozane is more stable to PDC hydrolysis than the predecessor β-lactam partner of tazobactam (i.e., piperacillin). Importantly, PDCs are not efficient at hydrolyzing ceftolozane (4). Unlike tazobactam, ceftolozane inhibits penicillin binding proteins (PBPs), allowing tazobactam to target other serine β-lactamases (e.g., TEM-1) and ESBLs (e.g., CTX-M-15) that are often present in P. aeruginosa. Alarmingly, resistance to new antibiotics is often reported before FDA approval (5). Indeed, resistance to ceftolozane-tazobactam has already emerged and may prove to be a significant clinical threat (6–8).

**FIG 2:**
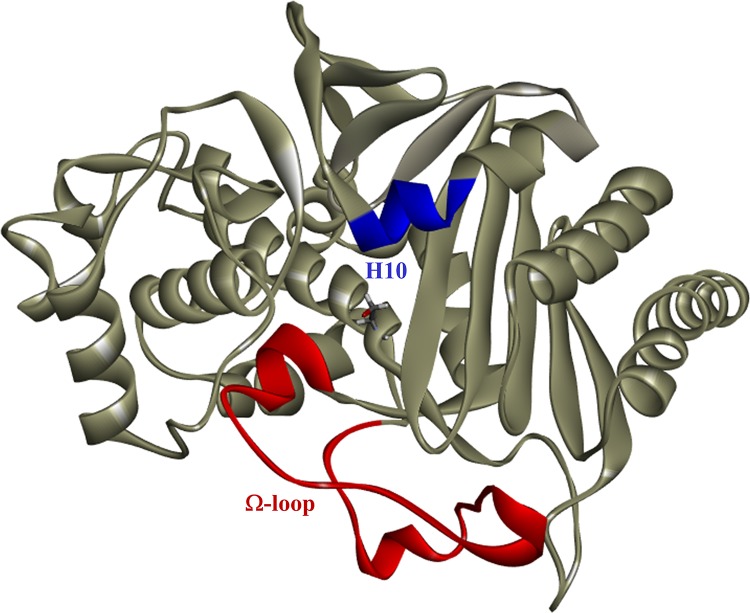
PDC structure (PDB ID 4HEF) showing the Ω-loop (red) at the entrance of the active site and the H10 helix (blue) of the R2 region that accommodates the R2 side chain of cephalosporins. The active site serine is shown in stick model representation.

Among the resistance mechanisms is the evolution of the PDC β-lactamase amino acid variants that often increase catalysis and spectrum of cephalosporin hydrolysis. Conserved motifs within the AmpC β-lactamases, largely the Ω-loop and H10 helix of the R2 region, seem particularly prone to amino acid substitutions (Fig. 2), insertions, and deletions that enlarge the active site and accommodate bulkier R1 and R2 groups of the β-lactam, respectively (9, 10). Several PDC Ω-loop variants (PDC-50 V213A; PDC-74, -75, and -78 G216R; PDC-79 and -86 E221K; PDC-80 E221G; and PDC-85 Y223H) identified originally in highly drug-resistant P. aeruginosa clinical isolates conferred resistance to a panel of antibiotics, including ceftazidime and ceftolozane-tazobactam (11, 12). Herein, we assessed the contribution of these residues (V213, G216, E221, and Y223) in PDC-3 toward ceftolozane-tazobactam and ceftazidime resistance by performing a structure-function analysis. In addition, the E221K variant was selected for in-depth biochemical analysis, revealing robust hydrolysis of ceftolozane as well as ceftazidime. Importantly, we find that avibactam restored in the strains expressing the variants susceptibility to ceftolozane and ceftazidime. The boronic acid transition state inhibitors (BATSIs) also lowered ceftolozane and ceftazidime MICs of strains harboring the variants, but the E221K strain maintained modestly high MICs (8 to 16 mg/liter). Our analysis presented herein provides the first biochemical and molecular dynamics rationale for enhanced catalytic action against this novel 3′ aminopyrazolium cephem.

## RESULTS AND DISCUSSION

### Single amino acid substitutions in the Ω-loop of PDC-3 enhance resistance toward ceftolozane and ceftazidime.

Previously, five different amino acid substitutions in the Ω-loop (V213A, G216R, E221K, E221G, and Y223H) of the PDC β-lactamase were found to confer enhanced β-lactam resistance in clinical isolates (12). Consistent with this finding, a 5-amino-acid-residue deletion, which included E221 and Y223, and a 7-amino-acid-residue deletion, including V213 and G216, in PDC β-lactamases was concomitant with increased resistance to ceftazidime-avibactam (13). In response to selective pressure using ceftolozane-tazobactam, the evolution of the E221K (E247K including the signal peptide sequence) emerged in a *Pseudomonas* strain (11). This particular amino acid substitution arose in SRT-1 of Serratia marcescens as early as 20 years ago (9, 14). To study the role of each of these residues in the mechanism of resistance and anticipate therapeutic approaches against the variants, we engineered the V213A, V213G, G216A, G216R, E221A, E221K, E221G, Y223A, and Y223H variants in PDC-3 for susceptibility determinations, followed by a focused analysis of the E221K variant’s interactions with ceftolozane and ceftazidime.

The susceptibility of the variants expressed in an isogenic Escherichia coli DH10B background was tested against a panel of representative cephalosporins, penicillins, monobactams, and carbapenems (Table 1). Amino acid substitutions in PDC-3 led to the most significant impact on MICs against ceftolozane, ceftazidime, and aztreonam. Interestingly, the structures of ceftolozane, ceftazidime, and aztreonam contain the “bulkiest” and most hydrophilic R1 groups of the compounds tested (Fig. 1A). The dimethylacetic acid in the R1 group in ceftazidime (also found in ceftolozane and aztreonam) was specifically associated with antipseudomonal activity (15). The E221K variant conferred the greatest resistance with an increase of 128-fold in the ceftolozane MIC and an increase of 32-fold in ceftazidime MIC relative to that of the wild-type strain (Table 1).

**TABLE 1:** Susceptibility results of various β-lactams against PDC-3 and the variants expressed in the pBC SK (-) vector in *E. coli* DH10B cells

Species and strain^*a*^	MIC value (mg/liter)							
	Ceftolozane	Ceftazidime	Cefotaxime	Cefepime	Ampicillin	Piperacillin	Aztreonam	Imipenem
P. aeruginosa								
18SH	8	64	>256	128	4,096	512	128	2
PAO1	0.5	1	16	4	2,048	4	4	2

E. coli								
DH10B	0.5	0.25	<0.06	0.125	4	2	0.125	0.5
DH10B pBC SK	0.5	0.25	<0.06	<0.06	4	2	0.125	0.25
DH10B pBC SK *bla*_PDC-1_	0.5	2	8	0.125	256	16	0.5	0.25
DH10B pBC SK *bla*_PDC-3_	0.5	2	8	0.06	256	16	1	0.25
DH10B pBC SK *bla*_PDC-3 V213A_	4	32	16	0.125	1,024	64	8	0.5
DH10B pBC SK *bla*_PDC-3 V213G_	4	16	16	0.125	1,024	64	8	0.5
DH10B pBC SK *bla*_PDC-3 G216R_	8	32	16	0.25	1,024	16	16	0.5
DH10B pBC SK *bla*_PDC-3 G216A_	2	16	8	0.125	1,024	64	8	0.5
DH10B pBC SK *bla*_PDC-3 E221K_	64	64	4	0.25	256	16	8	0.5
DH10B pBC SK *bla*_PDC-3 E221G_	16	32	8	0.125	512	64	4	0.5
DH10B pBC SK *bla*_PDC-3 E221A_	2	8	16	0.125	1,024	128	4	0.5
DH10B pBC SK *bla*_PDC-3 Y223H_	8	16	8	0.125	512	32	4	0.5
DH10B pBC SK *bla*_PDC-3 Y223A_	32	32	16	0.25	256	16	4	0.5

a *P. aeruginosa* 18SH and *P. aeruginosa* PAO1 are control strains expressing PDC-3 and PDC-1, respectively.

### Avibactam and boronic acid transition state inhibitors (BATSIs) overcome resistance to ceftazidime.

Several β-lactam–β-lactamase inhibitor combinations were selected to test the efficacy of compounds from three different classes of inhibitors against the strains expressing the variant PDCs. Susceptibility was tested against tazobactam, a first-generation sulfone inhibitor, combined with piperacillin or ceftolozane; avibactam (16), a diazabicyclooctane (DBO) combined with ceftazidime; and two boronic acid transition state inhibitors (BATSIs) (a cephalothin analog S02030 [17] and a ceftazidime analog LP-06 [18]) (see Fig. 1B for structures), each combined with ceftazidime.

In the piperacillin-tazobactam and ceftolozane-tazobactam combinations, tazobactam led to a 2- to 8-fold attenuation of the variant strain MICs compared to those of the single antibiotic (Table 2). The E221K variant conferred the greatest resistance to ceftolozane and ceftazidime. Susceptibility to ceftolozane and ceftazidime was substantially restored when these oxyimino cephalosporins were paired with avibactam (MICs ≤ 1 mg/liter). In other studies, select Ω loop variants were associated with resistance to these clinically available combination therapies (12, 13). Therefore, we extended the study to include testing of BATSI inhibitors, which are chemically distinct from tazobactam and avibactam (Fig. 1). Interestingly, the LP-06 and S02030 BATSIs lowered the MIC values of ceftolozane and ceftazidime for all variant strains, but the strain expressing E221K maintained the highest MICs (8 to 16 mg/liter) (Table 2). LP-06 lowered the MICs more effectively than S02030 did, particularly for the Y223A variant strain. Further, LP-06 was as potent as avibactam in all variant strains except the strain harboring the E221K variant.

**TABLE 2:** Susceptibility results of various β-lactams compared to β-lactams in combination with β-lactamase inhibitors against PDC-3 and variants expressed in the pBC SK (-) vector in *E. coli* DH10B cells

Species and strain^*a*^	MIC value (mg/liter)^*b*^										
	PIP	PIP- TAZ	TOL	TOL- TAZ	TOL- AVI	TOL- LP06	TOL- S02030	CAZ	CAZ- AVI	CAZ- LP06	CAZ- S02030
P. aeruginosa											
18SH	512	256	8	8	8	8	8	64	2	32	64
PAO1	4	4	0.5	0.5	0.5	0.5	0.5	1	1	0.5	1

E. coli											
DH10B	2	2	0.5	0.25	0.5	0.5	0.25	0.25	0.25	≤0.25	0.25
DH10B pBC SK	2	2	0.5	0.25	0.25	0.25	0.25	0.25	0.25	≤0.25	0.25
DH10B pBC SK *bla*_PDC-1_	16	8	0.5	0.5	0.5	0.25	0.25	2	0.25	0.25	0.25
DH10B pBC SK *bla*_PDC-3_	16	4	0.5	0.5	0.5	0.25	0.25	2	0.25	0.25	0.25
DH10B pBC SK *bla*_PDC-3 V213A_	64	16	4	2	0.5	0.25	0.25	32	0.25	0.25	1
DH10B pBC SK *bla*_PDC-3 V213G_	64	8	4	2	0.5	0.25	0.25	16	0.25	0.25	1
DH10B pBC SK *bla*_PDC-3 G216R_	16	8	8	4	0.25	0.5	0.5	32	0.25	0.25	1
DH10B pBC SK *bla*_PDC-3 G216_	64	8	2	2	0.25	0.25	0.25	16	0.25	0.25	0.25
DH10B pBC SK *bla*_PDC-3 E221K_	16	8	64	8	1	8	8	64	0.5	8	16
DH10B pBC SK *bla*_PDC-3 E221G_	64	16	16	4	0.5	1	2	32	0.5	0.5	1
DH10B pBC SK *bla*_PDC-3 E221_	128	16	2	2	0.5	0.5	0.25	8	0.25	0.25	0.25
DH10B pBC SK *bla*_PDC-3 Y223H_	32	4	8	1	0.25	0.5	0.5	16	0.5	0.5	2
DH10B pBC SK *bla*_PDC-3 Y223A_	32	4	32	4	1	2	8	32	0.5	0.5	8

a *P. aeruginosa* 18SH and *P. aeruginosa* PAO1 are control strains expressing PDC-3 and PDC-1, respectively.

b PIP, piperacillin; TAZ, tazobactam; TOL, ceftolozane; AVI, avibactam; CAZ, ceftazidime.

### Protein expression of PDC-3 and the E221K variant in the cell is lower than the other variants.

**FIG 3:**
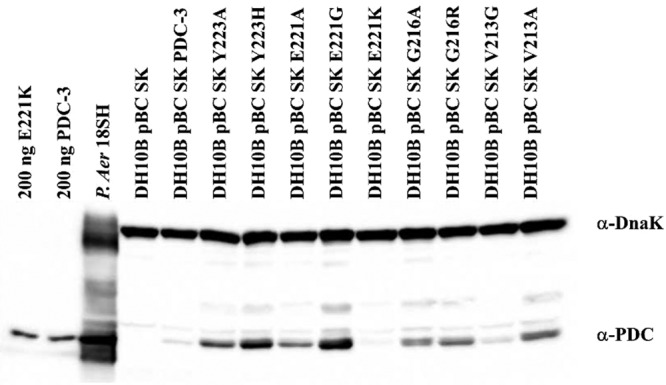
Immunoblots of E. coli DH10B whole-cell lysates of PDC-3 and variants expressed in the pBC SK (-) vector. The P. aeruginosa 18SH clinical isolate and purified PDC-3 β-lactamase are used as positive controls. An anti-DnaK antibody was used as a loading control.

To determine whether the elevated MIC values for the E221K variant strain were due to a higher expression level of the β-lactamase protein in the cell, we developed and used an anti-PDC-3 antibody to perform immunoblotting on the strains harboring PDC-3 and the variants (Fig. 3). E. coli strains variably expressed each PDC-3 variant. On the basis of the epitope mapping of PDC-3 using a SPOT-synthesis technique, we posit that changes in the binding of the anti-PDC-3 antibody to the newly introduced amino acids in the variants does not account for differences in intensities in the immunoblot; the epitopes do not encompass the V213, G216, E221, and Y223 amino acid residues (Fig. S1). Strikingly, the amount of the E221K variant most closely mimicked the low expression of wild-type PDC-3 β-lactamase. Further, the E221K variant was detected at the lowest level of expression relative to the other variants, a remarkable observation when considering that the E221K variant confers the highest level of resistance against the tested antibiotics (Tables 1 and 2). Supporting this observation, previous studies suggested that overexpression of PDC proteins alone is not always sufficient to account for such robust resistance (11, 12, 19). To further explore the mechanisms of resistance, we tested the β-lactam kinetic characteristics of the E221K variant.

10.1128/mBio.02085-18.1 FIG S1 (A) SPOTs membrane of the PDC-3 protein identifying eight major immunogenic epitopes for recognition by the anti-PDC-3 antibody. (B) Mapping of the epitopes (purple) on the X-ray crystal structure model of PDC-1 (PDB ID 4GZB) with the T105A and catalytic S64 positions identified. Download FIG S1, TIF file, 1.9 MB. Copyright © 2018 Barnes et al. 2018 Barnes et al. This content is distributed under the terms of the Creative Commons Attribution 4.0 International license.

### Mechanisms of catalysis: PDC-3 β-lactamase fails to establish an acyl complex with ceftolozane but forms a prolonged acyl-complex with ceftazidime.

**FIG 4:**
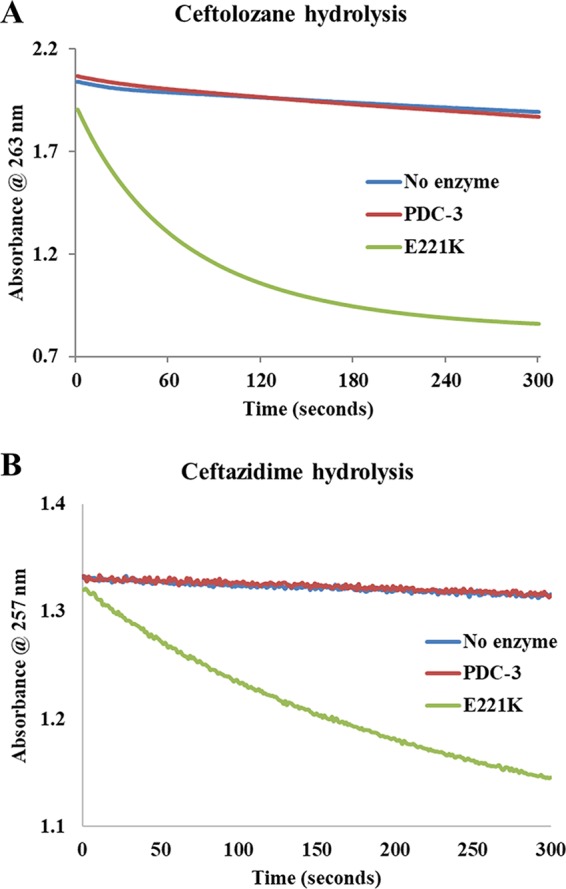
Hydrolysis of ceftolozane (A) and ceftazidime (B) by PDC-3 and the E221K variant. Ceftolozane (100 μM) was tested with 1 μM enzyme (PDC-3 or E221K). Ceftazidime (300 μM) was assayed with 100 nM enzyme (PDC-3 or E221K).

The wild-type PDC-3 hydrolyzed ceftolozane and ceftazidime albeit poorly (Fig. 4A and B), consistent with the MIC results. As expected, PDC-3 demonstrated a lower *K_m_* toward ceftazidime compared to ceftolozane. Using a competitive inhibition assay with nitrocefin as the reporter substrate, the *K_i_*_app_ of ceftolozane (1.3 mM) was determined to be 60-fold higher than that of ceftazidime (22 µM) with PDC-3. Thus, PDC-3 interacts favorably with ceftazidime compared to ceftolozane, yet is not hydrolyzed (Fig. 4B).

**FIG 5:**
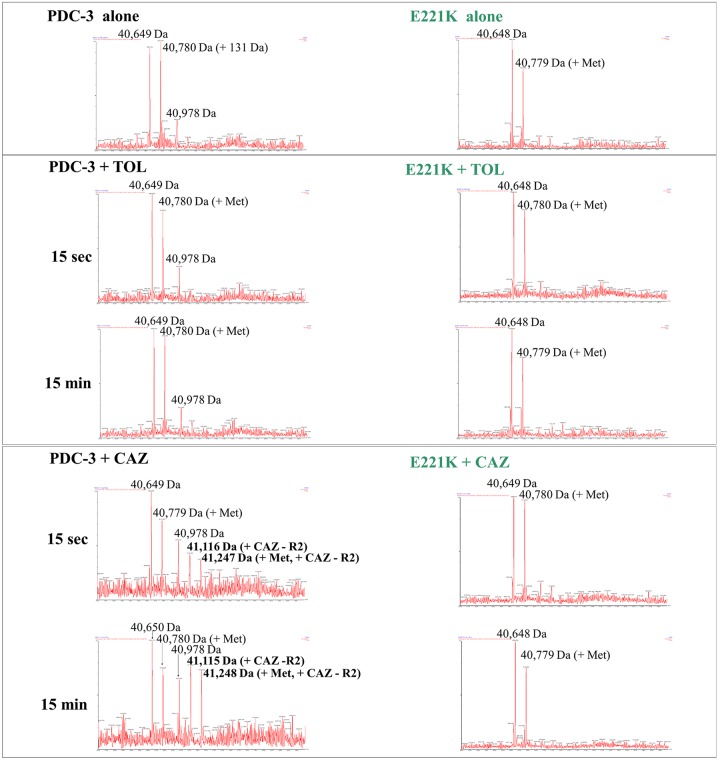
Electrospray ionization mass spectrometry (ESI-MS) of pure PDC-3 and the E221K variant incubated with ceftolozane (TOL) (MW, 666 Da; MW without R2 group, 471 Da) or ceftazidime (CAZ) (MW without R2 group, 468 Da) for 15 s or 15 min at a 1:1 molar ratio. Methionine (Met) has a molecular weight of 131 Da. Mass accuracy is ±5 Da.

To further investigate the catalytic mechanism, we performed timed mass spectrometry. Two isoforms of PDC-3 and E221K were expressed and purified due to the presence of additional methionine at the N terminus (20) (Fig. 5). Using a 1:1 molar ratio of enzyme to substrate, timed mass spectrometry analysis captured the binding of PDC-3 to ceftazidime, but not to ceftolozane (Fig. 5 and Table 3). The observed masses reflect cephalosporins without the R2 group (circled in Fig. 2). Cephalosporins that demonstrate efficient R2 elimination typically have more reactive β-lactam rings and enhanced activity (21). Elimination of the R2 group from cephalosporins results from electron transfer upon binding to β-lactamases and was previously shown for ceftazidime and ceftolozane among other cephalosporins (21–25). An acyl-complex with ceftolozane or ceftazidime was not detected with the E221K variant, but ceftazidime without its R2 group was captured in complex with PDC-3 (Fig. 5).

**TABLE 3:**
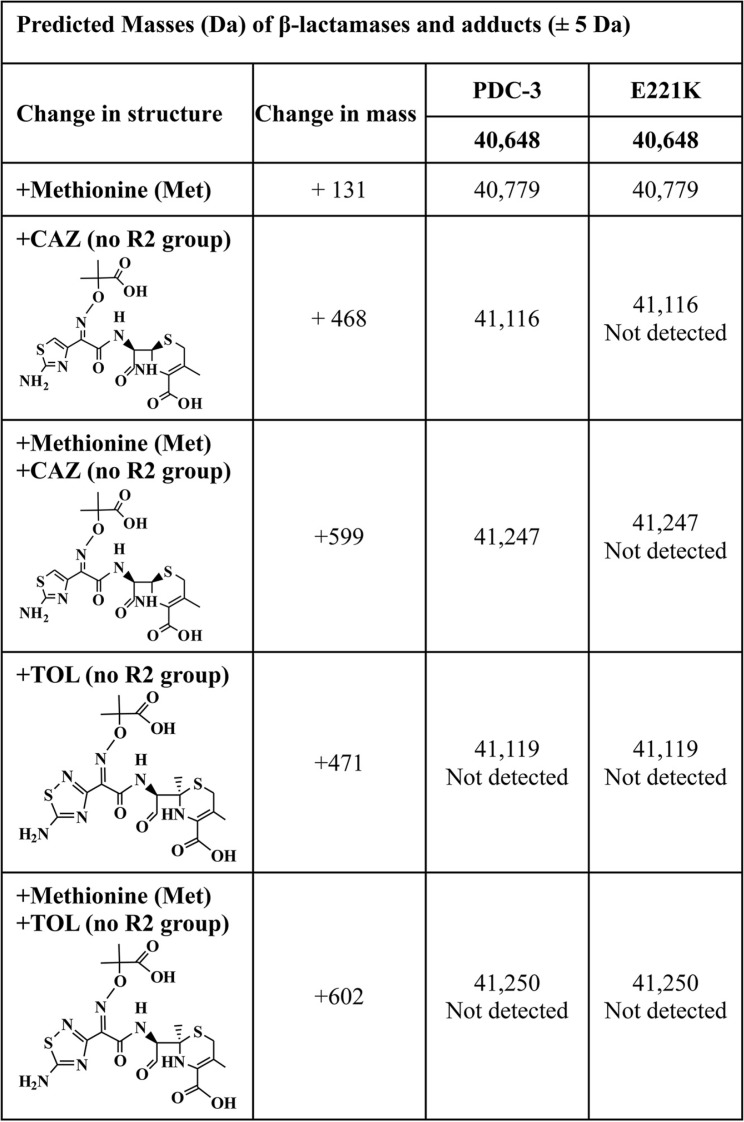
Predicted masses of PDC-3 and the E221K variant β-lactamases with and without the addition of ceftazidime and ceftolozane^*a*^

a Predicted masses of PDC-3 and the E221K variant β-lactamases with and without the addition of ceftazidime (CAZ) and ceftolozane (TOL). The R2 group is eliminated from the cephalosporin upon binding to β-lactamase. The additional Met residue was a result of cloning and does not impact β-lactamase activity.

As indicated by a lower *K_m_*, nearly 60-fold-lower apparent inhibition constant (1.3 mM ceftolozane *K_i_*_app_ versus 22 µM ceftazidime *K_i_*_app_), and the ability to capture the ceftazidime acylated PDC-3 complex, ceftazidime must be able to occupy the active site of PDC-3 despite overall hydrolytic deficiency. Contrary to ceftazidime, the failure of PDC-3 to hydrolyze ceftolozane is more likely due to the inability of the PDC-3 active site to accommodate the more basic and extended 3′ aminopyrazolium-containing R2 side chain of ceftolozane (Fig. 2) and form an acyl-complex (see “Molecular modeling” below). We also have not ruled out product inhibition as a mechanism. Modifications of the 3-aminopyrazolium side chains during the structural derivation of ceftolozane correlated with stability to class C β-lactamases, likely due to steric hindrance (26). The mechanisms of these third-generation cephalosporins contrast with that of the fourth-generation cephalosporin cefepime, which has a zwitterionic quaternary amine as the R2 group that decreases affinity for β-lactamases (Fig. 1) (27) and effectively overcomes resistance conferred by the PDC variants in E. coli (Table 1).

### Acquisition of hydrolytic efficiency: the E221K variant demonstrates robust hydrolysis of ceftolozane and ceftazidime.

Consistent with increased resistance of the variants compared to PDC-3 (Table 1 and 2), the E221K variant acquired an ability to efficiently hydrolyze ceftolozane (Fig. 4A) and ceftazidime (Fig. 4B). *K_m_* for the E221K variant for ceftazidime is 174 ± 20 µM (Table 4), considerably higher than previously reported values of 35 µM (28) and 51.7 ± 2.1 µM (20) for PDC-3. Turnover (*k*_cat_) for E221K for ceftazidime is 9 ± 1 s^−1^ (Table 5), substantially greater than the 0.02 s^−1^ (28) and 0.002 s^−1^ (4) values reported for PDC-3. Catalytic efficiency (*k*_cat/_*K_m_*) for E221K (5.2 ± 0.8 × 10^−2 ^µM^−1^ s^−1^) is notably higher for ceftazidime than the reported values of PDC-3; 0.0006 µM^−1^ s^−1^ (28) and 0.00033 µM^−1^ s^−1^ (4).

**TABLE 4:** Steady-state kinetics of the E221K variant with ceftazidime and ceftolozane^*a*^

Parameter	E221K-ceftolozane	E221K-ceftazidime
*V*_max_ (µM/s)	1.0 ± 0.1	0.9 ± 0.1
*K_*m*_* (µM)	341 ± 64	174 ± 20
*k*_cat_ (s^−1^)	10 ± 1	9 ± 1
*k*_cat_/*K_*m*_* (µM^−1^ s^−1^)	(2.9 ± 0.6) × 10^−2^	(5.2 ± 0.8) × 10^−2^

a PDC-3 hydrolysis of ceftolozane and ceftazidime was negligible.

**TABLE 5:** Inhibition kinetics of PDC-3 and the E221K variant with the BATSI compounds^*a*^

β-Lactamase (IC_50_)	IC_50_ (nM) for β-lactamase with:	
	LP-06	S02030
PDC-3 (1.5 nM)	9 ± 1	62 ± 2
E221K (20 nM)	342 ± 20	769 ± 30

a Inhibition kinetics of PDC-3 and the E221K variant with the BATSI compound LP-06 or S02030, determined after a 5-min preincubation with LP-06 or S02030 with cell lysate. Nitrocefin was used as the reporter substrate.

E221K exhibited a higher *K_m_* value for ceftolozane (341 ± 64 µM) than for ceftazidime (174 ± 20 µM) (Table 4). The other kinetic parameters were similar for E221K between the substrates: ceftolozane (*k*_cat_ of 10 ± 1 s^−1^ and *k*_cat_/*K_m_* of 0.029 ± 0.006 µM^−1^ s^−1^) and ceftazidime (*k*_cat_ of 9 ± 1 s^−1^ and *k*_cat_*/K_m_* of 0.052 ± 0.008 µM^−1^ s^−1^) (Table 4). The efficient hydrolysis of ceftolozane and ceftazidime by E221K is consistent with the inability to capture the acyl-E221K complexes by timed mass spectrometry due to the rapidity of the reaction (Fig. 5).

### The BATSIs are potent inhibitors of PDC-3 and the E221K variant.

The steady-state inhibitory kinetics of the BATSIs were assessed using nitrocefin as a reporter substrate (nitrocefin *K_m_* for PDC-3 (20 ± 2 µM) and E221K (17 ± 3 µM)). S02030, the cephalothin analog, and LP-06, the ceftazidime analog, inhibit PDC-3 and the E221K variant at nanomolar concentrations. LP-06 and S02030 inhibited PDC-3 more efficiently than E221K did (Table 5). Comparison between the two inhibitors suggests that LP-06 has superior inhibitory activity compared to S02030 for both PDC-3 and E221K (Table 5).

### BATSI inhibitors increase the thermodynamic stability of PDC-3 and E221K.

**FIG 6:**
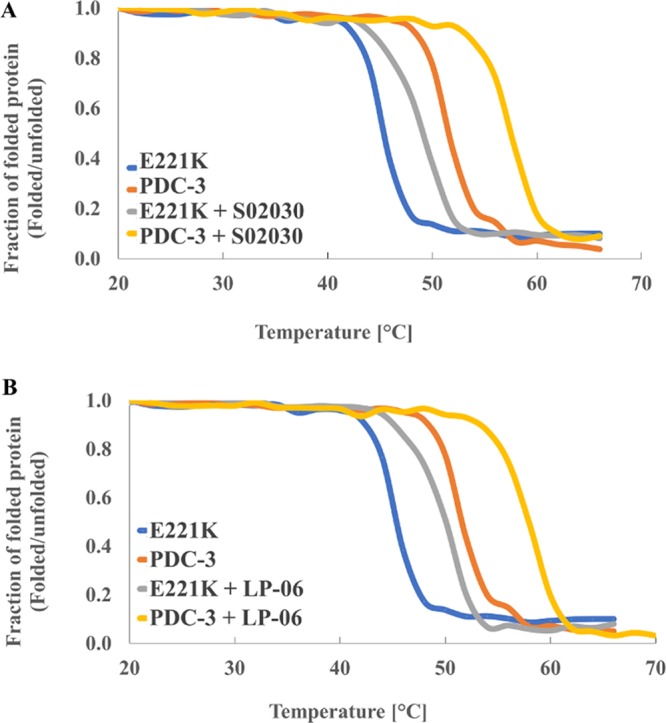
Thermal denaturation of PDC-3 and E221K with S02030 (A) or LP-06 BATSI (B) inhibitors, expressed as a ratio of folded protein to unfolded protein.

The thermodynamic stability of PDC-3 and E221K was determined from equilibrium unfolding curves (Fig. 6). Thermal denaturation revealed that the E221K variant is less stable compared to PDC-3 (*T_m_* of 45°C and 52°C, respectively), with a ΔΔGu = 2.32 ± 0.2 Kcal/mol loss in stability (Fig. 6). Binding of the S02030 BATSI inhibitor to either PDC-3 or the E221K variant increased the stability (6°C increase for PDC-3 and 3 to 4°C increase for the variant) (Fig. 6A). Similarly, the LP-06 BATSI stabilized both PDC-3 (6°C increase) and E221K (5°C increase) (Fig. 6B), as previously observed for other BATSIs with SHV-1, ADC-7, and AmpC (29–32). Increased stability between the inhibitors and the β-lactamases is indicative of higher energy for noncovalent interaction in the covalent acyl complexes compared to the β-lactamase alone (31). Thus, increased stability of PDC-3 with the BATSIs, reflective of increased interactions and the formation of inhibitory complexes, would predict the lower ceftazidime-BATSI MICs compared to ceftazidime alone MICs in Table 2.

Notably, the thermal denaturation profiles showed a further distinction between PDC-3 and the E221K variant. The transition where the inhibitor-bound PDC-3 began to unfold occurred at 4°C, higher than for PDC-3 alone, shifting the curve for the inhibitor-bound PDC-3 to the right (Fig. 6). After the initial transition, PDC-3 unfolded at a similar rate with and without inhibitor as reflected by a uniform difference (∼4°C) between the two melting curves (Fig. 6). Conversely, the E221K variant started to unfold at the same temperature regardless of whether it was bound by inhibitor, but the steeper slope of E221K alone suggested a higher rate of unfolding in the absence of inhibitor.

**FIG 7:**
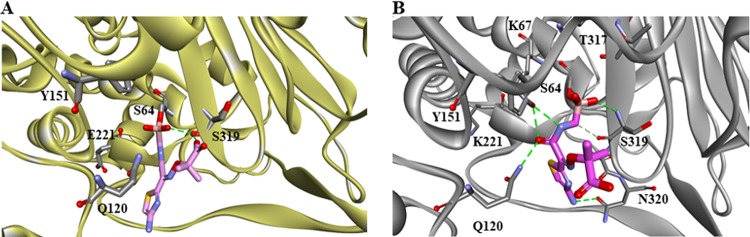
Molecular modeling of PDC-3 (A) and the E221K variant (B) bound to LP-06, a ceftazidime-like BATSI. LP-06 is represented as a stick figure with carbon in pink, sulfur in yellow, nitrogen in purple, oxygen in red, and boron in rose color.

The lower unfolding rate of inhibitor-bound E221K compared to inhibitor-bound PDC-3 could be due to additional interactions between the LP-06 and E221K as suggested by our molecular modeling (Fig. 7). The boronic and carboxylic acids of LP-06 interact with residue S319 in PDC-3. In the E221K variant, the interaction between the boronic acid and S319 is maintained, but the carboxylic acid-S319 bond is lost. Further, LP-06 forms new bonds with the S319, N320, Q120, and Y151 residues of E221K. To further explore the structural changes in the E221K compared to PDC-3, we employed molecular modeling and molecular dynamic simulations using ceftolozane as the substrate.

### Molecular dynamic simulations reveal conformational changes in the E221K variant that accommodate binding of ceftolozane.

**FIG 8:**
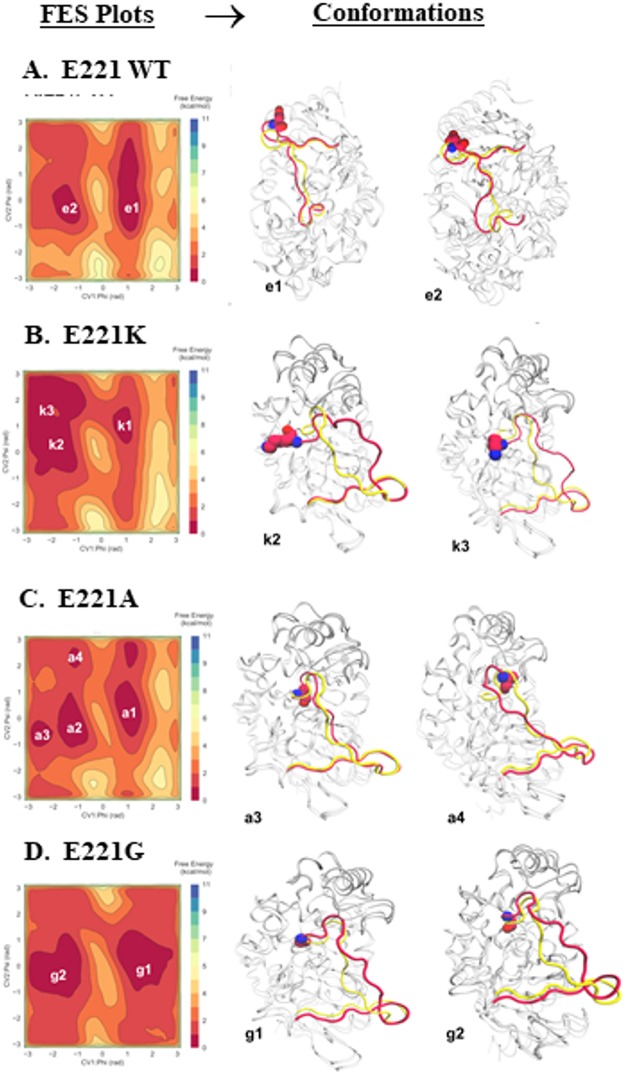
Conformational free energy surface (FES) reconstructed from metadynamics simulations of PDC-3 (A) and the E221 variants E221A (B), E221K (C), and E221G (D). Representative conformations corresponding to each energy basin (e.g., e1, e2) are superimposed onto the crystal structure, shown on the right. The Ω-loop of the crystal structure of wild-type E221 (yellow) and the simulated result of the structure (red) are highlighted. The amino acid of interest (wild-type E221 or the A, K, and G substitutions) in each panel is visualized in red and blue using a Corey-Pauling-Koltun (CPK) representation.

Classical atomistic molecular dynamics (MD) and well-tempered metadynamics simulations (WT-MetaD) were carried out to enhance conformational sampling of PDC-3 and variants (Fig. 8). Free energy surface (FES) plots were calculated for each system, highlighting basins of lowest energy states as shown in the red wells in Fig. 8 (e.g., e1 and k1), wherein each basin corresponds to an ensemble of conformations of the protein. Conformations of PDC-3 and the variants were extracted from the free energy minima and superimposed onto the crystal structure (PDB ID, 4HEF, yellow).

The E221K variant adopts conformations in the free-energy landscape that are not accessible by PDC-3 (E221) (Fig. 8). Generally, the E221 variants are able to explore the conformation in k1, a1, and g1 energy basins that are similar to the conformation in the wild-type (e1) (Fig. 8B to D). However, other energy basins such as k3 are observed exclusively by the E221K variant (Fig. 8B). Moreover, the clustered (representative) structures illustrate that the conformation adopted by the Ω-loop in particular is distinct from that of the crystal structure. Consistent with the conformational changes, the hydrogen bonding patterns are altered in the E221 variants. In PDC-3, the side chain of E221 forms hydrogen bonds with K67, but these bonds are disrupted in the E221K, E221A, and E221G variants.

### In the Michaelis-Menten complex, rotation of the Y223 residue in E221K supports binding of ceftolozane.

**FIG 9:**
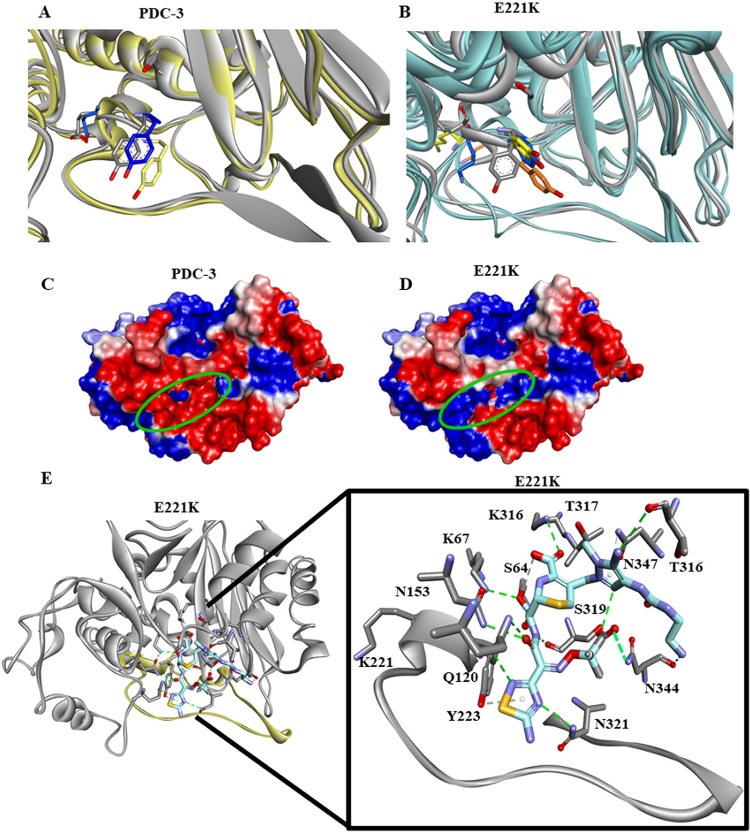
Representative conformations of PDC-3 (A) and E221K (B) corresponding to individual energetic basins extracted from metadynamics simulations which show the conformational change of Y223. Connolly representation of PDC-3 (C) and the E221K variant (D) based on the calculated electrostatic potential showing significant electrostatic changes introduced by the E221K substitution. (E) Molecular modeling of E221K and ceftolozane as acyl enzyme complex. Ceftolozane is represented as a stick figure with carbon in blue, sulfur in yellow, nitrogen in purple, and oxygen in red.

Enhanced sampling simulations identified conformations in the E221K variant, which were not found in the wild type. The conformations identified for E221K indicated a rotation of the Y223 side chain. The Y223 residue in PDC-3 adopts several positions within the same plane (Fig. 9A), whereas in the E221K variant, Y223 flips into a perpendicular plane, opening a hidden cavity adjacent to the catalytic site (Fig. 9B), which is occluded by Y223 in the crystalline form of PDC-3. These data are supported by previous work in E. coli class C cephalosporinases, where a steric clash between the tyrosine ring and R1 cephalosporin side chains was concluded from the crystal structure of AmpC with a glycine substitution in the Y223-equivalent residue (Y221G) of PDC (33). Increased flexibility in the Ω-loop was also observed in the crystal structure of the E221K analog of AmpC of E. coli (E219K) (33). This newly adopted conformation and change in the architecture of the Ω-loop facilitate the formation of Michaelis-Menten complex between the E221K variant and ceftolozane. The aminopyrazole group in the R2 side chain of ceftolozane seems to be the specific moiety responsible for its inability to bind to PDC-3. These data are supported by measurable steady-state kinetic parameters (e.g., *K_m_* of 341 ± 64 µM) (Table 4) and elevated MICs (ceftolozane MIC PDC-3 of 0.5 µM → E221K 64 µM) (Table 2).

### In the acyl-complex, ceftolozane interacts favorably with the E221K variant, but it is unable to interact productively with PDC-3.

Connolly representations depict overt changes in electrostatic potential when the E221 residue is changed to a lysine (Fig. 9C and D). Y223 and E221 are located in the center of the green circle in Fig. 9C and D. The calculated potential of PDC-3 is 1318 kT compared to the potential of the E221K variant at 1,304 kT (delta =13.4 kT). Docking of ceftolozane into the active site of the E221K variant illustrates that ceftolozane forms hydrogen bonds with residues S64, N153, K316, S319, K67, N344, and N321 (Fig. 9E). In addition, ceftolozane forms steric interactions with Y223, S319, and N347.

An accurate acyl-enzyme model of ceftolozane and PDC-3 was not possible because ceftolozane is too bulky to readily position into the active site of PDC-3. Ceftolozane sterically clashes with the active site residues at the entrance of PDC-3 (positions 120 to 125) and Ω-loop residues (positions 209 to 217), giving rise to a multitude of unproductive conformations. To better understand the effect of the single amino acid substitutions at an atomic level, we analyzed the dynamics and energetics of the PDC-3 and the E221K variant.

Enhanced sampling simulations reveal that the ion-pair interactions made between D191 and K226 help anchor the Ω-loop in a conformation that allows the side chain of Y223 to change its orientation to point away from the catalytic site. This in turn reveals a hidden pocket, which can accommodate the tail of ceftolozane. The increase in conformational flexibility and expansion of the active site entrance likely explains why the E221K variant acquires the ability to accommodate ceftolozane in its active site and hydrolyze both ceftolozane and ceftazidime, despite the difference in size (Fig. 2).

### Conclusions.

Our previous findings showed that amino acid substitutions in the C_3_/C_4_ β-lactam carboxylate recognition region of PDC-3 increase or maintain antibiotic susceptibility (20). The emergence of mutations in AmpC β-lactamases as a mechanism of resistance to ceftolozane has gone largely unexplored (from a biochemical and mechanistic standpoint) with an underestimated occurrence of ≥1.5% resistance to β-lactams (12), but it has attracted more attention in recent years (11, 12, 34, 35). Here, we focused on clinically important amino acid substitutions in the Ω-loop of PDC-3 and how they contribute to the resistance profile. While PDC-3 is our primary focus in this study, the vital impact that the residues in the Ω-loop have on structural integrity and catalytic activity is not limited to PDC-3, or even class C, β-lactamases (22, 36–41). Herein, we first showed that the hydrolysis of ceftazidime by the wild-type β-lactamase PDC-3 is deficient despite the ability of ceftazidime to dwell in the active site of PDC-3. To our knowledge, this insight has not been significantly appreciated. A single amino acid substitution, E221K, in PDC-3 enabled the β-lactamase to acquire increased hydrolytic activity and induced multiple conformations of the variant, identified by enhanced sampling simulations. This particular substitution has already evolved in the clinic in multiple species (12, 14), and therefore may be evolutionarily favored to emerge in additional drug-resistant pathogens. The potential severity of the variants in the clinic is exacerbated by increased resistance to aztreonam as well as ceftolozane and ceftazidime. Further, employment of molecular dynamic simulations and other biophysical methods to capture dynamic interactions are useful for elucidating protein structure-function relationships.

The gain of function observed in enhanced catalysis is not obtained without a sacrifice. The E221K protein acquires hydrolytic activity at the detriment of conformational restraints that destabilize the infrastructure of the protein, which is partially restored by binding to inhibitors. Importantly, despite the E221K variant’s enhanced cephalosporinase activity, avibactam and the LP-06 BATSI effectively lowered ceftolozane and ceftazidime MIC values of the E221K strain. BATSIs and DBOs may be able to serve as partners for novel combinational therapies against P. aeruginosa infections. More importantly, by differing significantly from the ceftolozane-tazobactam mechanism of inactivation, these “second-generation” β-lactamase inhibitors offer promise against this significant threat to our armamentarium (42).

## MATERIALS AND METHODS

### Critical reagents and strains.

Ceftazidime was procured from Sigma-Aldrich (catalog no. C3809) and Research Products International (catalog no. 33527) and used interchangeably throughout the experimentation. Merck & Co., Inc. (Kenilworth, NJ, USA) provided ceftolozane powder. Avibactam was purchased from Advanced ChemBlocks (catalog no. R16073). Piperacillin (catalog no. P8396), cefotaxime (catalog no. C7912), and chloramphenicol (catalog no. R4405) were purchased from Sigma-Aldrich. Imipenem was obtained from USP (catalog no. 1337809) and from the commercial source. Tazobactam (catalog no. 15141) and aztreonam (catalog no. 15151) were purchased from Chem-Impex International. Ceftolozane-tazobactam, cefepime, and meropenem were obtained from their commercial sources. Nitrocefin (catalog no. BR0063G) was purchased from Oxoid. The LP-06 and S02030 BATSIs were synthesized as previously described (43, 44). See Drawz et al. (20) for a discussion on the P. aeruginosa control strains (18SH AmpC for PDC-3 and PAO1 for PDC-1).

### Expression and purification of PDC-3 and E221K.

PDC-3 β-lactamase was cloned into the pER24a (+) vector as previously described (20). PDC-3 β-lactamase was purified from E. coli Origami 2 DE3 (Novagen) cells carrying the pET24a (+) *bla*_PDC-3_ or pET24a (+) *bla*_PDC-3-E221K_ plasmid that produces the corresponding PDC-3 protein without its signal peptide, similar to the previously described protocol (20, 45). Cells were grown in 500 ml super optimal broth (SOB) at 37°C to an optical density at 600 nm (OD_600_) of approximately 0.6 to 0.8 and induced with 1 mM isopropyl-β-D-1-thiogalactopyranoside (IPTG) for a minimum of two and a half hours to express the β-lactamase. The cell pellets were generated by centrifugation and frozen at −20°C for ≥12 h prior to a 40-min lysis in 50 mM Tris-HCl buffer (pH 7.4) containing 40 mg/ml lysozyme, 0.1 mM magnesium sulfate, 250 U benzonase nuclease, and 1 mM ethylenediaminetetraacetic acid (EDTA). The supernatant was further purified using a HiTrap SP Sepharose cation exchange column (GE Healthcare Life Sciences) and 25 mM Tris-HCl (pH 7.4). Protein was eluted from the column using 25 mM Tris-HCl (pH 7.4) and 0.5 M NaCl. If needed, the sample of protein was concentrated using centrifugal filter units with a 10,000-dalton-molecular-weight cutoff (Millipore). The protein was subjected to gel filtration using a Sepahadex (75 prep grade) HiLoad 16/600 column in 10 mM phosphate-buffered saline (PBS), pH 7.4. The final sample of protein was concentrated using centrifugal filter units with a 10,000-dalton-molecular-weight cutoff (Millipore). The purity of the proteins was assessed by quadrupole time of flight (Q-TOF) mass spectrometry (see below). Protein concentrations were determined by measuring absorbance at a wavelength of 280 nm (λ_280_) and using the protein’s extinction coefficient (Δε = 54,320 M^−1 ^cm^−1^) obtained using the ProtParam tool at ExPASy Bioinformatics Resource Portal.

### Purification of anti-PDC-3 antibody.

For the SPOTS membrane, the PDC-3 protein (3 mg of protein in 10 mM phosphate-buffered saline [PBS], pH 7.4) was provided to New England Peptide (Gardner, MA) for polyclonal antibody production in a rabbit. The serum was purified using a protein G column to obtain polyclonal immunoglobulin antibodies against PDC-3. Briefly, 3 ml of rabbit serum was added to a binding buffer composed of 20 mM NaH_2_PO_4_ (pH 7.0) and added to a 5-ml HiTrap Protein G HP column (GE Healthcare, Piscataway, NJ) with a flow rate of 0.5 ml/min. The bound anti-PDC-3 was eluted from the column using 0.1 M glycine-HCl (pH 2.7) and neutralized with 1 M Tris-HCl (pH 9.0). The antibody concentration was quantified using spectrophotometric determination at 280 nm and stored at −20°C.

### Epitope mapping (SPOTs membrane).

An epitope map of the PDC-3 β-lactamase was created using SPOT synthesis on a cellulose membrane by JPT Peptide Technologies GmbH (Berlin, Germany) as previously described (46, 47). Briefly, peptides with an acetylated N terminus were covalently bound to a Whatman 50 cellulose membrane by the C terminus. The membrane was incubated in methanol for 5 min, washed three times with Tris-buffered saline (TBS) for 10 min each time, and blocked with 5% milk in TBS overnight. The following day, the membrane was washed four times with TBS for 4 min each time. Primary antibody was added at 1 mg/liter in 5% milk in TBS for 3 h. Blots were washed four times with TBS-Tween (TBS-T) for 10 min each time before incubation with a secondary antibody (protein G-HRP) diluted 1:5,000 for 1 h. Blots were washed four times with TBS-T for 10 min each time and processed with the ECL Prime kit (Amersham/GE Healthcare, Piscataway, NJ).

### Immunoblotting of PDC-3 β-lactamase.

Log-phase protein expression was determined as previously described (45). One milliliter of bacteria grown to an OD_600_ of 0.6 to 0.8 in lysogeny broth (LB) in 20 µg/liter chloramphenicol was pelleted and resuspended in sodium dodecyl sulfate-polyacrylamide gel electrophoresis (SDS-PAGE) buffer. Each sample (10 µl) was separated by SDS-PAGE. Proteins were transferred to polyvinylidene fluoride membranes for 2 h at 80 V. The membranes were blocked overnight in 5% milk in Tris-buffered saline (TBS) solution, followed by a 1- to 3-h incubation with a 0.1 mg/liter primary PDC-3 (anti-18SH, 42) rabbit polyclonal antibody and an anti-DnaK rabbit polyclonal antibody (Stressgene, San Diego, CA) diluted 1:15,000 (loading control) in 5% milk/TBS and finally incubated with the protein G-HRP conjugate at a 1:10,000 dilution for 1 h in 5% milk/TBS. An ECL Prime Western blot exposure kit from Amersham was used to visualize the blot on the Fotodyne imager.

### Susceptibility testing.

Variant genes were synthesized into the pBC SK (-) vector by Celtek Bioscience (Franklin, TN). Mueller-Hinton (MH) agar-dilution MIC measurements were performed against isogenic transformants producing PDC-3 and nine variants of PDC-3 in pBC SK (-) according to the Clinical and Laboratory Standards Institute (CLSI) guidelines as previously described (39, 48). Avibactam, LP-06, and S02030 were tested at a constant 4 µg/ml in combination with various concentrations of ceftazidime. Piperacillin-tazobactam was maintained at a ratio of 8 piperacillin to 1 tazobactam. Ceftolozane-tazobactam was maintained at a ratio of 2 ceftolozane to 1 tazobactam. The MICs are reported as the concentrations at which bacterial growth was no longer observed. All MIC measurements were performed at least three times.

### Steady-state kinetic and inhibitor analysis.

Steady-state kinetic and inhibitor parameters were determined by using an Agilent 8453 diode array spectrophotometer at room temperature as previously described (20, 43, 49). Each assay was performed in 10 mM phosphate-buffered saline (PBS) at pH 7.4 at room temperature (RT = ∼25°C) in a quartz cuvette with a 1-cm path length. Measurements were obtained using ceftolozane and ceftazidime. The kinetic parameters, *V*_max_ and *K_m_*, were obtained with a nonlinear least-squares fit of the data (Henri Michaelis-Menten, equation 1) using Origin 7.5VR (OriginLab, Northampton, MA).

(1) $$v=\frac{V_{max\;[S]}}{K_{m}+\left[S\right]}$$

For the reversible boronic acid inhibitors, measurements were obtained for E221K and PDC-3 using nitrocefin (Δε482 = 17,400 M^−1^ cm^−1^) as a reporter substrate. Because of the time-dependent inhibition observed previously with some of the chiral boronates (43), the BATSIs were preincubated with enzyme for 5 min in phosphate-buffered saline before initiation of the reaction with the addition of substrate. The initial velocity (0 to 10 s) was measured in the presence of a constant concentration of enzyme (1.5 nM PDC-3 and 20 nM E221K) and increasing concentrations of the inhibitors against the fixed concentration (100 μM) of the indicator substrate, nitrocefin. The IC_50_ is the concentration of inhibitor that reduces the velocity by 50%. IC_50_ values were determined by fitting the initial velocity (*v_0_*) measurements to equation 2.

(2) $$v_{0}=\;\frac{V_{max[S]}}{K_{m}\left[1+\;\frac{I}{{IC}_{50}}\right]+[S]}$$

For competitive inhibition assays used to determine *K_i_*_app_, 150 μM nitrocefin was used as the reporter substrate with increasing concentrations of ceftazidime or ceftolozane. Initial velocities were plotted against concentrations of ceftazidime or ceftolozane. *K_i_*_app obs_ were determined by dividing the slope by the *y* intercept. *K_i_*_app_ was calculated by correcting *K_i_*_app obs_ for nitrocefin affinity and concentration (equation 3).

(3) $$K_{i\;app}=K_{i\;app\;obs}/[1+([NCF]/K_{m\;NCF})$$

### Electrospray ionization mass spectrometry (ESI-MS).

Five micrograms of β-lactamase (PDC-3, E221K) was incubated with the substrate at a 1:1 molar ratio (PDC-3 and ceftazidime or ceftolozane) in 10 mM PBS at pH 7.4 for a total reaction volume of 20 μl for the times indicated in the figures. Reactions were quenched with 10 μl acetonitrile and added to 1 ml of 0.1% formic acid in water. Samples were analyzed using Q-TOF Waters Synapt-G2-Si and Waters Acquity UPLC BEH C_18_ 1.7-μm column (2.1 by 50 mm). MassLynx V4.1 was used to deconvolute protein peaks. The tune settings for each data run were as follows: capillary voltage at 3.5 kV, sampling cone at 35, source offset at 35, source temperature of 100°C, desolvation temperature of 500°C, cone gas at 100 liters/h, desolvation gas at 800 liters/h, and nebulizer bar at 6.0. Mobile phase A was 0.1% formic acid in water. Mobile phase B was 0.1% formic acid in acetonitrile. The mass accuracy of this system is ±5 Da.

### Thermal denaturation.

Circular dichroism (CD) experiments were carried out in a Jasco (Easton, MD) J-815 spectrometer with a Peltier effect temperature controller. Quartz cells with a 0.1-cm path length were used for all experiments.

PDC-3 enzyme and the E221K variant (10 μM) with or without S02030 or LP-06 (200 μM) were monitored for thermal denaturation by CD at λ_215_ and λ_221_ between 20 and 80°C with a heating rate of 2°C/min. Two-state behavior was indicated by identical curves at each wavelength. Raw equilibrium denaturation data were normalized to the fraction of denatured protein (*f_U_*). With the assumption of a reversible two-state transition (N ↔ U), equilibrium constants (*K*_eq_) at any given temperature were calculated, as previously described (32), using equation 4

(4) $$K_{eq}=f_{U}/(1-f_{U})$$

With the assumption that enthalpy and entropy changes do not vary with the temperature (Δ*C_p_* = constant), from the Gibbs free energy and van’t Hoff equation (Δ*G* = Δ*H*_VH_ − *T*Δ*S*= −*RT*ln*K*_eq_) (50) the Δ*H*_VH_ was determined as the slope from equation 5 (*R* is the gas constant). The melting temperature *T_m_* was determined at the midpoint of equilibrium folding (*T* = *T_m_*) when Δ*G* = 0.

(5) $$\ln\;K_{eq}=1/T\;(-\Delta H/R)+\Delta S/R$$

The free energy of unfolding was determined as previously described using the equation ΔΔ*G_u_* = Δ*T_m_*Δ*S_u_*^PDC-3^ (Schellman), where Δ*S_u_*^PDC-3^ = 0.326 ± 0.02 Kcal mol^−1^ K^−1^.

### Molecular modeling. (i) Docking.

Structural representations of PDC-3 and the E221K variant of KPC-2 β-lactamase were generated using the crystal coordinates of P. aeruginosa AmpC (PDC-1) (PDB ID 4HEF) and Discovery Studio 2016 (DS 2016) (Dassault Systèmes BIOVIA; Discovery Studio Modeling Environment, San Diego, CA) molecular modeling software as previously described (37, 51). The T79A substitution (T79A substitution corresponds to T105A in 4HEF because of the 26-amino-acid signal peptide) was incorporated into the PDC-1 β-lactamase structure to obtain the PDC-3 β-lactamase structure.

### (ii) Acyl complex.

The PDC-3 β-lactamase structure and the variant model were solvated and minimized to an RMS of 0.03 Å using the conjugate gradient method. The intact and acylated ceftolozane were constructed using the Fragment Builder tools and minimized using a Standard Dynamics Cascade protocol of DS. Both molecules were docked automatically into the active site of the PDC-3 and E221K variant using CDOCKER module of DS. To obtain acyl-enzyme complexes, the most favorable conformation demonstrating anticipated active site contacts was chosen, and the complex was further minimized. The spatial distribution of the electrostatic potential and the potential of PDC-3 and E221K variant atoms were calculated using the DelPhi program with Debye-Huckel boundary condition approximation. The DelPhi calculation was based on a two-dielectric implicit solvent model and a finite difference method to solve the Poisson-Boltzmann equation on a cubic grid (52, 53).

### Classical molecular dynamics and well-tempered metadynamics.

The structure of the PDC-3 β-lactamase was prepared starting from the Protein Data Bank (PDB ID 4HEF) (54) with a T79A substitution. Nine variants (V213A, V213G, G216A, G216R, E221A, E221G, E221K, Y223A, and Y223H) were constructed *in silico* using the ICM mutagenesis program (55). The system was prepared using a high-throughput molecular dynamics (HTMD) protocol (56). Amberff14SB force field was used to define the parameters of the protein (57), combined with explicit TIP3P water molecules (58). The systems were minimized with 1,000 steps of steepest descent integrator and equilibrated in the NPT ensemble for 5 ns, using a Berendsen barostat at 1 atm (59). The temperature was kept at 300 K° by a Langevin thermostat. A 550-ns production run was carried out for all the systems in the NVT ensemble with a time step of 4 fs. All the simulations were carried out with the ACEMD program (60). To enhance sampling, Well-Tempered Metadynamics simulations (61, 62) (WTMetaD) starting from the equilibrated structures of the wild-type PDC-3 and variant structures were performed at 300 K using the software ACEMD and the PLUMED 1.3 plug-in using an integration step of 4 fs. To study the flexibility of the Ω-loop as a function of the variants, we chose the dihedral angles of the WT/variant residue to be the collective variable (CV). The choice of the CVs (CV1 = ϕ and CV2 = ϕ of the substituted residue) was based on the observation that the slowest motions in a protein are a function of their backbone flexibility (63). Therefore, the differences in the structural effects resulting from the changes between the wild type and the variants should be pronounced in the backbone dihedral angles. The bias was added on the two CVs by setting Gaussian width of 0.1 and 0.1 rad, respectively, while the Gaussian height was at 0.5 kJ/mol. Gaussians were deposited every 4 ps, so that the deposition rate was equal to 0.125 kJ/(mol·ps). The bias factor was fixed to 15. A total of 550 ns in the NVT ensemble were needed to reach full convergence of the free energy (see Fig. S2 in the supplemental material). The free energy surface of the WTMetaD simulation as a function of the two CVs is readily obtained by integrating the deposited energy bias along the trajectory. The error on the minima and barriers of the free energy surface was estimated from the largest variation observed in the monodimensional projections along the collective variables during the last 100 ns of the simulation. It amounts to 0.5 kcal/mol.

10.1128/mBio.02085-18.2 FIG S2 Convergence of Well-Tempered metadynamics simulations for wild-type E221 (A) and the E221 variants (B to D). Diffusibility of CV1 (ϕ) (i) and CV2 (ψ) (ii) over simulation time. Projection of the free energy (FE) on collective variables CV1 (ϕ) (iii) and CV2 (ψ) (iv). (v) Gaussian height (kJ/mol) versus simulation time was plotted for each E221 variant. The other simulations show a similar behavior. Download FIG S2, TIF file, 2.6 MB. Copyright © 2018 Barnes et al. 2018 Barnes et al. This content is distributed under the terms of the Creative Commons Attribution 4.0 International license.

The structures corresponding to the minima were selected from the WTMetaD trajectories based on the values of the collective variables CV1 and CV2. The RMSF of the Ω-loop was calculated from the unbiased simulations of 1,000 ns of the WT and mutants, using the g_rmsf tool of the GROMACS 5.1.4 package (64). To remove the effect of slow, large-scale conformational transitions, the RMSF was calculated on the Cα atoms in overlapping windows of 100 ns each and averaged afterwards. The first 150 ns of the simulation were considered equilibration time and omitted from the calculation. The structural figures were generated using VMD and ICM-Pro software (55, 65, 66).
