# Supplementary figures and images for: Deciphering the Evolution of Cephalosporin Resistance to Ceftolozane-Tazobactam in Pseudomonas aeruginosa
Source: mBio. 2018 Dec 11;9(6):e02085-18. doi: 10.1128/mBio.02085-18 (PMC6299481; doi:10.1128/mBio.02085-18)

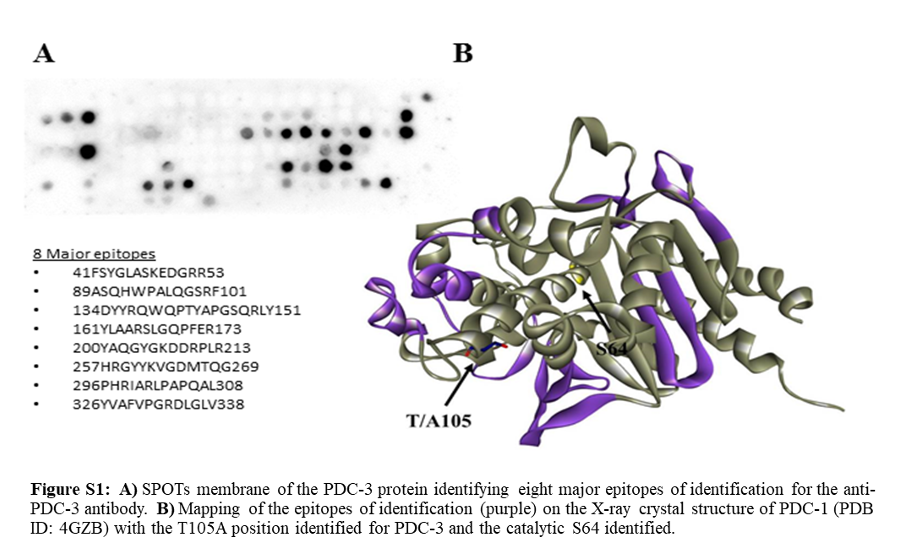

Supplement: FIG S1 [file mbo006184188sf1.tif]

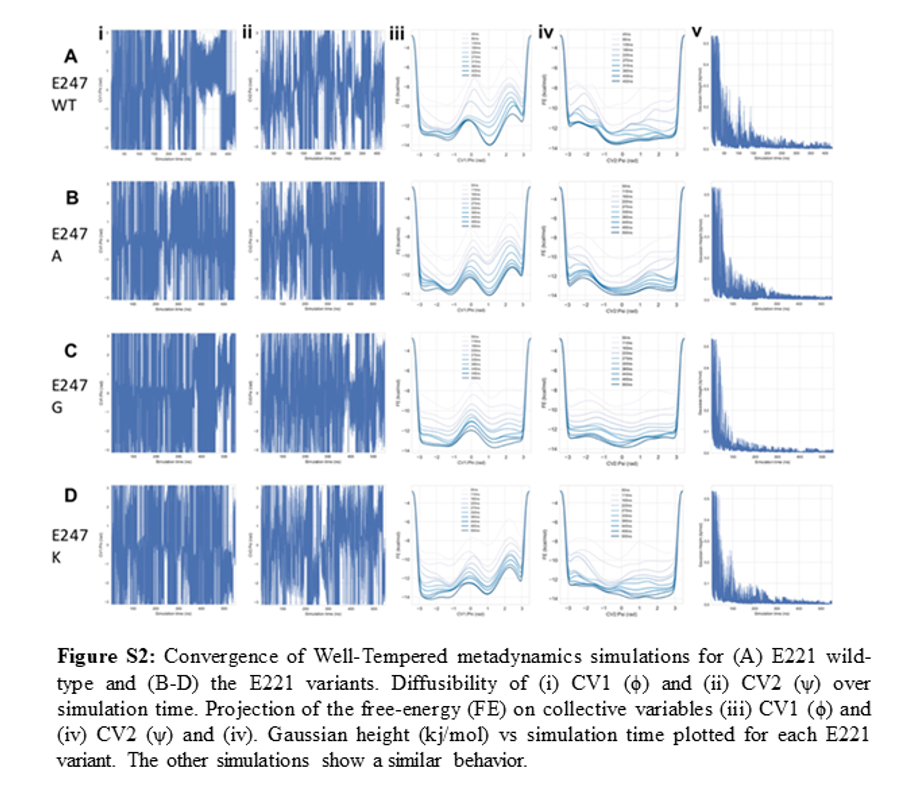

Supplement: FIG S2 [file mbo006184188sf2.tif]
